# Reliability and validity of general health questionnaire (GHQ-12) for male tannery workers: a study carried out in Kanpur, India

**DOI:** 10.1186/s12888-017-1253-y

**Published:** 2017-03-21

**Authors:** Gyan Chandra Kashyap, Shri Kant Singh

**Affiliations:** Department of Mathematical Demography and Statistics, International Institute for Population Sciences, Govandi Station Road, Deonar Mumbai, 400088 India

**Keywords:** Cronbach’s alpha, Confirmatory factor analysis, GHQ-12, Internal consistency, Reliability, Tannery, Validity

## Abstract

**Background:**

The purpose of this study was to test the reliability, validity and factor structure of GHQ-12 questionnaire on male tannery workers of India. We have tested three different factor models of the GHQ-12.

**Methods:**

This paper used primary data obtained from a cross-sectional household study of tannery workers from Jajmau area of the city of Kanpur in northern India, which was conducted during January–June, 2015, as part of a doctoral program. The study covered 286 tannery workers from the study area. An interview schedule containing GHQ-12 was used for tannery workers who had completed at least 1 year at their present occupation preceding the survey. To test reliability, Cronbach’s alpha test was used. The convergent test was used for validity. Confirmatory factor analysis was used to compare three factor structures for the GHQ-12.

**Results:**

A total of 286 samples were analyzed in this study. The mean age of the tannery workers in this study was 38 years (SD = 1.42). We found the alpha coefficient to be 0.93 for the complete sample. The value of alpha represents the acceptable internal consistency for all the groups. Each item of scale showed almost the same internal consistency of 0.93 for the male tannery workers. The correlation between factor 1 (Anxiety and Depression) and factor 2 (Social Dysfunction) was 0.92. The correlation between factor 1 (Anxiety and Depression) and factor 3 (Loss of confidence) was the highest 0.98. Comparative fit index (CFI) estimate best-fitted for model-III that gave the CFI value 0.97. The SRMR indicator gave the lowest value 0.031 for the model-III.

**Conclusions:**

The findings suggest that the Hindi version of GHQ-12 is a reliable and valid tool for measuring psychological distress in male tannery workers of Kanpur city, India. Study found that the model proposed by the Graetz was the best fitted model for the data.

## Background

The General Health Questionnaire (GHQ)-12 is a well-known and efficient tool for measuring the mental health status of the respondent subjects. The GHQ-12 scale is used worldwide in different segments of practice and research — clinical, epidemiological and psychological [1, 2]. When it was first introduced, the General Health Questionnaire contained 60 items in the scale, which were subsequently reduced to 30, then 28 items and, finally, to just 12 items [3, 4] in its present form.

GHQ-12 is the most refined scale for measuring the psychological well-being of various segments of the human population [5]. Many studies have validated GHQ-12 tool as a reliable measure of mental health in different segments of populations across countries [6–20].

It is evident from the study of a systematic review across occupational and population based studies provides the comparative results for occupational and population based study for GHQ-12 scale. Results from the study indicate a higher prevalence of mental health for the occupational based studies in comparison of population based studies [21].

Questionnaire consists of 12 items with each item measuring the severity of mental health problems in the 4 weeks preceding the study. Each item was assessed on a four-point scale (less than usual, no more than usual, rather more than usual, much more than usual). In this study GHQ-12 used the four-point Likert scale (1 to 4). The minimum possible total score is 12 and maximum, 48. The positive items are recoded from 1 (not at all) to 4 (much more than usual) and negative items from 4 (not at all) to 1 (much more than usual). As the application of GHQ-12 in any research setting is well-documented, it was decided to translate it into Hindi. (Hindi language).

Tanneries have attained considerable notoriety for the polluting nature of their work and the serious occupational health risks like mental health disorder, respiratory problems, musculoskeletal disorder, injuries etc. that they pose. Male tannery workers are constantly exposed to many harmful chemicals and physical hazards. Male tannery workers usually involved in many hazardous work process loading and unloading of raw hides, transferring the work, lifting the raw hides etc. in tannery premises. There is high risk of developing mental health disorder in such working conditions. The poor and unsafe working conditions act as stressors and increase the susceptibility of the male tannery workers. This study utilized the Hindi version of GHQ-12 scale to measure the mental health status of male tannery workers of Kanpur city, India. In present study we have tested GHQ-12 scale for the tannery workers, because they work in very hazardous work environment along with involvement in chromium exposure during tanning process, leather dust, exposure to chemical agents, ergonomic stressor increases their susceptibility which can be linked to mental health problem. So for, this study has tested the reliability, validity and factor structure of GHQ-12 scale for male tannery workers.

## Methods

Data for the present research was drawn from a cross-sectional household study of tannery workers in the Jajmau area of Kanpur City in the state of Uttar Pradesh, India. The study was conducted during the period January–June, 2015, and was part of a PhD program. All total 286 tannery workers from the study area were interviewed.

A three-stage sampling design was used. In the first stage, we selected seven areas in which most households have members working in the tanneries of nearby Jajmau. In second stage, three areas were selected on the basis probability proportion to size (PPS) i.e., the highest number of households with tannery workers in the area. In the third stage, we listed the households in which at least one member was a tannery worker. If a household was found to have more than one member working in tanneries, we selected the respondent through KISH table. In this manner, 100 samples were selected from each area through systematic random sampling. The three selected areas were Budhiyaghat, Tadbagiya and Ashrafabad in the Jajmau suburban part of Kanpur city.

GHQ-12 English version scale had been translated into Hindi version by following the standard procedure. Intensive pre-testing was done with the tannery workers of Jajmau area for testing the internal consistency the GHQ-12 tool of Hindi version. Before starting the interviews, we have explained about the purpose of the survey and requested to participate in the survey. After that face-to-face interviews were conducted among those who agreed to participate by using a structured pre-tested questionnaire on the tannery workers. Suitable statistical techniques were used to meet the objectives of study. The correlation matrix was constructed to understand the correlation between the items of GHQ-12. The item-scale analysis of GHQ-12 was also performed. Cronbach alpha test was used to estimate the reliability (internal consistency) of GHQ-12 for the male tannery workers who participated in the study.

A large number of studies has used confirmatory factor analysis for estimating the factor structure of the GHQ-12 scale. This study had verified three different models. Model 1 was unidimensional. Model II contained the two factors: the first factor had all six positively worded items, and factor two had all six negatively worded items [22]. Model III include three factors: Anxiety and depression, Social Dysfunction and Loss of confidence [23]. The present study has used the maximum likelihood method for estimating the factor loading for the three different models. To test the goodness of fit, we have assessed the following indices; RMSEA: Root mean squared error of approximation, AIC: Akaike’s information criterion, BIC: Bayesian information criterion, CFI: Comparative fit index, TLI: Tucker-Lewis index, SRMR: Standardized root mean squared residual.

### Description of the indices

#### RMSEA

The RMSEA estimate the parameter of the model that fit the population’s covariance matrix. Presently RMSEA was known as one of the most informative fit indices due to its sensitivity to the number of estimated parameters in the model. The cut-off value of RMSEA estimates varies from 0.6 to 0.7 within this range model is acceptable or best fit [24].

#### AIC

AIC considered as the first model criterion. Currently, it is most widely used model selection tool. AIC used to delineate between different fitted models having the same dimension [25].

#### BIC

In the computation of BIC based on the empirical log-likelihood and does not require the specification of priors. Basically, BIC has an asymptotic approximation to a transformation of the Bayesian posterior probability of a model [26].

#### CFI

Comparative fit index measures the fit of the model even when the sample size is small. The values are closer to 1 indicating the suitable model. But, cut off criterion of CFI ≥ 0.90 is acceptable. At present CFI is one of the most popularly fit indices because measures least affected by sample size [24, 27, 28].

#### TLI

TLI an index that prefers the simpler models. This index is well accepted where sample size is very small, and it gives the most accurate results. Due to the non-normed characteristics of this index value which can exceed above 1.0, which create some problem in the interpretation of the result. The cut-off point is 0.80 for this index while researcher suggested TLI ≥ 0.95 the threshold level [25].

#### SRMR

The SRMR describe the square root of the difference between the residuals of the sample covariance matrix and the hypothesized covariance model. The value of SRMR ranges 0 to 1 for fitting the model. The value 0 indicates the perfect fit and values as high as 0.08 are acceptable [26].

### Participants

The study comprised 286 male tannery workers of age group 18–70 years from Jajmau in suburban Kanpur.

## Results

### Descriptive findings

Table 1 presents the descriptive findings of the study. The mean age of tannery workers was 38 (SD = 1.42) years. Around 66% of the tannery workers were illiterate and only 11% had studied up to high school and above. The majority (89%) of tannery workers were on temporary job contracts, and their mean work experience in their present workplaces was 10 (SD = 0.92) years. They were involved in different types of work in the tanneries they were working in: beam house work (8.42% of the workers), wet finishing (24.21%), dry finishing (50.53%) and miscellaneous (16.84%). The respondent tannery workers also reported that they worked almost every day in the week with 9 to10 hours as the mean working day. Mean number of working days was 6.50 (SD = 0.06) and duration of a working day was 9.54 (SD = 0.19) hours.

**Table 1:** Socio-economic and work-related characteristics of tannery workers in Kanpur city, India, 2015

Variables	Tannery workers (%)	(Numbers, N)
Age in years^a^	38.55 ± 1.42	286
Education		
Illiterate	66.32	189
Up to primary	13.33	38
Middle school	8.77	25
High school & above	11.58	33
Work experience in current tannery^a^	10.10 ± 0.92	285
Work experience in previous tannery^a^	7.95 ± 1.25	99
Job status		
Temporary job (daily wages)	89.12	254
Permanent job	10.88	31
Type of work		
Beam house work	8.42	24
Wet finishing work	24.21	69
Dry finishing work	50.53	144
Miscellaneous work	16.84	48
Average working hours in day^a^	9.54 ± 0.19	285
Average working days in a week^a^	6.50 ± 0.06	285
Religion		
Hindu	33.92	97
Muslim	66.08	189
Caste		
SC/ST	65.38	187
Other backward classes	18.53	53
Others	5.59	16
Don’t know	10.49	30
Exposure to the media		
No exposure	23.8	66
Any exposure	76.92	220

^a^Mean ± SD

About two-thirds of the workers were Muslims (66.08%) and one-third of them Hindus. The majority of the tannery workers (65.38%) belonged to SC/ST caste groups. Significantly, 6% belonged to the Other castes.

Table 2 presents the correlation matrix of unidirectional GHQ-12 items with inter-item reliability and the value of Cronbach’s α coefficient. The correlation matrix shows the statistical measure of association between the GHQ-12 items. Item 1 shows the high correlation with the item 3 and negative correlation with the item 6. Further, Item 6 show the negative correlation with the item 7, 8, 9, 10, 11, 12. Item 7 show the lowest correlation with item 9, and it was the highest with the item 12. Item 8 had the lowest correlation with the item 10. The inter-item correlation of GHQ-12 items was ranged from 0.49 to 0.53. And the average inter-item correlation was 0.50. Cronbach’s alpha test is one of the most commonly used tools to test reliability estimates and was employed in this study to test the internal consistency of GHQ-12 questionnaire for the male tannery workers. We found the alpha value to be 0.93 for the entire sample. The value of alpha represents the acceptable internal consistency for all the groups.

**Table 2:** Correlations between items in GHQ-12 scale, Inter-item reliability

	Item 1	Item 2	Item 3	Item 4	Item 5	Item 6	Item 7	Item 8	Item 9	Item 10	Item 11	Item 12	Inter-item Reliability^a^
Item 1	1												
Item 2	0.46	1.00											0.497
Item 3	0.73	0.50	1.00										0.523
Item 4	0.64	0.51	0.73	1.00									0.494
Item 5	0.49	0.60	0.50	0.48	1.00								0.506
Item 6	-0.70	-0.39	-0.70	-0.56	-0.38	1.00							0.524
Item 7	0.69	0.43	0.61	0.59	0.40	-0.60	1.00						0.510
Item 8	0.51	0.41	0.50	0.62	0.37	-0.49	0.56	1.00					0.503
Item 9	0.55	0.53	0.55	0.53	0.54	-0.50	0.44	0.30	1.00				0.530
Item 10	0.51	0.46	0.53	0.50	0.52	-0.48	0.48	0.29	0.64	1.00			0.505
Item 11	0.60	0.61	0.55	0.53	0.59	-0.47	0.55	0.39	0.69	0.66	1.00		0.512
Item 12	0.71	0.48	0.63	0.56	0.44	-0.62	0.69	0.41	0.57	0.43	0.53	1.00	0.504

^a^Average inter-item reliability: 0.50; Cronbach’s α coefficient: 0.93

### Confirmatory factor analysis

Table 3 shows goodness-of-fit statistics for the three models estimated in this study. Model 1 was unidimensional. Model II contained two factors, and Model III include three factors: Anxiety and depression, Social Dysfunction and Loss of confidence [23]. Confirmatory factor analysis gave the lowest RMSEA value in model-III although all three models estimated the RMSEA’s value more than 0.08. Results show the lowest AIC and BIC value in model-II, whereas it was the highest in model-I. Comparative fit index (CFI) estimate best-fitted model-III that gave the CFI value 0.97 along with CFI value 0.93 in model-II. In all the three models TLI estimates ranged 0.86–0.95. The cut-off point of TLI was 0.80, and the threshold level was 0.95 that is acceptable. The SRMR indicator gave the lowest value 0.031 for the model-III. The value close to 0 indicates the perfect fit and the value up to 0.08 are acceptable. The model-III was the best model fitted for the data that given the better estimates of four indicators than model-I and model-II.

**Table 3:** Goodness-of-fit of three confirmatory factor analysis models (*N* = 286)

Statistics	Model-I	Model-II	Model-III
RMSEA	0.127	0.171	0.091
AIC	7552.22	3786.02	4008.18
BIC	7687.23	3851.70	4073.86
CFI	0.892	0.930	0.973
TLI	0.865	0.884	0.955
SRMR	0.061	0.420	0.031

*RMSEA* Root mean squared error of approximation, *AIC* Akaike’s information criterion, *BIC* Bayesian information criterion, *CFI* Comparative fit index, *TLI* Tucker-Lewis index, *SRMR* Standardized root mean squared residual

**Fig. 1:**
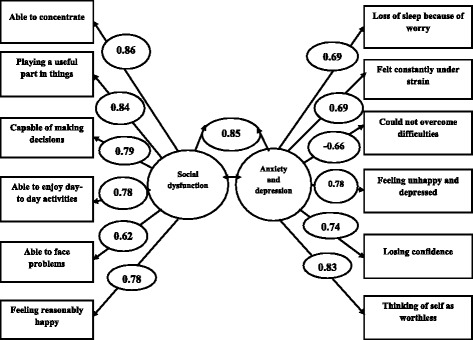
Standardized factor loadings and between-factor correlations between model I and model II

Figure 1 shows the standardized factor loadings and between factor correlations of positive worded (PW) items and negatively worded items (NW) in model II. The factor loading ranged between - 0.66-0.86. The positive worded (PW) items had higher correlations that range from 0.62–0.86 then negatively worded items and the correlation between negatively worded items ranged - 0.66- 0.83. Factor 1 includes all positively worded items and factor 2 includes all negatively worded items. The correlation between factor 1 (Social Dysfunction) and factor 2 (Anxiety and Depression) was 0.85.

**Fig. 2:**
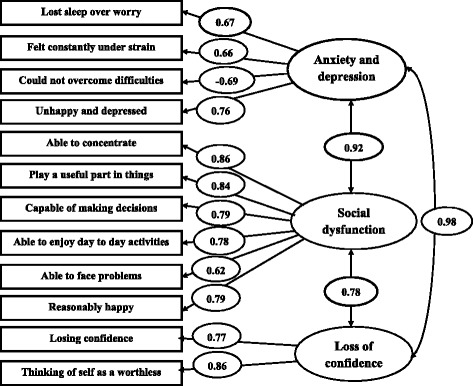
Standardized factor loadings and between-factor correlations. Boxes represent GHQ-12 items: one-way and two-way arrows indicate factor loadings between-factor correlations, respectively

Figure 2 shows the standardized factor loadings and between factor correlation of model III. The factor loading ranged between −0.69-0.98. The result shows the strong relationship between the three factors of the model III. The correlation between factor 1 (Anxiety and Depression) and factor 2 (Social Dysfunction) was 0.92. And, the correlation between factor 2 (Social Dysfunction) and factor 3 (Loss of confidence) were 0.78. The correlation between factor 1 (Anxiety and Depression) and factor 3 (Loss of confidence) was the highest 0.98. It is evident from the strong correlations between the three factors confirm that Graetz’s 3-factor model fitted the data in better than two other models in this study.

## Discussion

The GHQ-12 is the most refined and familiar scale for measuring the psychological well-being of diverse groups of people in different settings [6–20]. The GHQ-12 has been translated into several languages worldwide [7, 9, 13, 16–18, 20]. The aim of this study was to translate and test the reliability, validity and factor structure of GHQ-12 questionnaire for male tannery workers in India. The GHQ-12 scale is also acknowledged for its reliability in measuring the perceived general health status of employees working in different sectors [21, 29], which is a reason for its widespread acceptance. There are different strategies for estimating reliability. The commonly-used ones are test-retest reliability, equivalent (or parallel) forms reliability and internal consistency reliability [30, 31]. This study used the internal consistency reliability method.

There are different methods for testing reliability that are based on the theory of estimation of test reliability. They include Spearman-Brown prophecy formula, Kuder-Richardson formulas (KR-20 and KR-21), and Cronbach alpha, which is also called the alpha coefficient [32]. The Cronbach alpha test is more flexible because it can be applied when test item scores are dichotomous as well as when they are scored on the Likert scale. KR-20 or KR-21 methods can be applied only when test-item scores have dichotomous values. Therefore, this study used Cronbach alpha test for testing the reliability. An alpha test coefficient of 0.93 was obtained for the entire sample, which is a universally accepted value for confirming reliability [33]. We may then conclude that, for this study, this is a highly reliable and valid scale.

Confirmatory factor analysis was used to compare three-factor structures for the GHQ-12 scale. This study has estimated the indices RMSEA, AIC, BIC, CFI, TLI, and SRMR. The study has used a unidimensional model that is model-I, model-II contain the two factors: positively worded items and negatively worded items. The third model was proposed by the Graetz: anxiety and depression, social dysfunction and loss of confidence. Our results show that the unidimensional model was the poorest fit that evidence from the estimated indices. The two-factor model estimated some of the indicators which are in the acceptable range. Few studies had mentioned the two-factor model that is an artificial grouping of all positively worded items and all negatively worded items. The two-factor model also fit the model for selected indicators in the study. The three-factor model proposed by the Graetz that was the best-fitted model. The result shows that all the factor had the strong correlation with each other. The correlation between factor 1 and factor 2 was 0.92. And, the correlation between factor 2 and factor 3 was 0.78. The correlation between factor 1 and factor 3 was the highest 0.98. Several previous studies also documented the strong association between the three actors and best-fitted model in the different data sets [34–39].

## Conclusions

Our results showed that Hindi version of the GHQ-12 is a reliable and valid tool for measuring psychological distress among the male tannery workers. Our analysis of the three-factor model proposed by the Graetz found the best fit model; all three factors were strongly correlated with each other.

## Abbreviations

GHQ: General Health Questionnaire
PPS: Probability Proportional to Size
SC/ST: Schedule Caste/Schedule Tribes
SD: Standard Deviation
